# Supplementary material for: Relationship between youth cardiometabolic health and physical activity in medical records
Source: PLoS One. 2024 Jun 6;19(6):e0303583. doi: 10.1371/journal.pone.0303583 (PMC11156312; doi:10.1371/journal.pone.0303583)
Supplement: S1 Table — A1c- glycated hemoglobin; ALT—Alanine transaminase; HDL–high density lipoprotein; LDL–low density lipoprotein. (PDF) [file pone.0303583.s002.pdf]

S1Table. Guidelines used for normal and abnormal blood pressure and cardiometabolic lab values

Cardiometabolic risk factor	Normal range	Abnormal range	Implausible value
A1c	<5.7%	≥5.7%	<3%, >20%
ALT	≤25	>25	>500 IU/L
Blood pressure	based on child sex, age, and height percentile	Systolic or diastolic value >95th %ile	
HDL	45 – 150 mg/dL	≤45 mg/dL	<10mg/dL, >150 mg/dL
LDL	15 – 130 mg/dL	≥130 mg/dL	<15mg/dL, >200 mg/dL
Total cholesterol	50- 200 mg/dL	≥200 mg/dL	<50 mg/dL, >350mg/dL
Triglycerides	30 – 130 mg/dL	>130 mg/dL	<30 mg/dL, >1000 mg/dL

A1c- glycated hemoglobin; ALT - Alanine transaminase; HDL – high density lipoprotein; LDL – low density lipoprotein.
