# Supplementary material for: Relationship between youth cardiometabolic health and physical activity in medical records
Source: PLoS One. 2024 Jun 6;19(6):e0303583. doi: 10.1371/journal.pone.0303583 (PMC11156312; doi:10.1371/journal.pone.0303583)
Supplement: S2 File — (DOCX) [file pone.0303583.s004.docx]

**General Pediatrics Clinics Data: Tracking Obesity Prevalence and Prevention**

**Co-Investigator:**

Sarah Hampl, MD, Weight Management, Center for Children’s Healthy Lifestyles & Nutrition, Children’s Mercy Kansas City. 2401 Gillham Road. Kansas City, MO, 64108. 816-983-6764; [shampl@cmh.edu](mailto:shampl@cmh.edu)

**Co-Investigator:**
Jordan Carlson, PhD, Center for Children’s Healthy Lifestyles & Nutrition, Children’s Mercy Kansas City, 2401 Gillham Road, Kansas City, MO 64108. 816-234-9240; [jacarlson@cmh.edu](mailto:jacarlson@cmh.edu)

**Bethany Forseth, PhD,** Center for Children’s Healthy Lifestyles & Nutrition, Children’s Mercy Kansas City, 2401 Gillham Road, Kansas City, MO 64108 952-212-0497. Bhanson4@kumc.edu

**Study Coordinator:** Amy Papa, MS

Please see the MARS application for additional staff not listed on the protocol.

**Study Site(s):** Don Chisholm Center. 610 East 22^nd^ Street, Kansas City, MO 64108

**Protocol Version:**  (*4.0)* **Protocol Date:** (*4/2/2019)*

1. STUDY OBJECTIVES/HYPOTHESIS

Primary Objective(s)

Aim 1. To longitudinally track patient eating and activity behavior assessment results and related provider counseling in children presenting for well child care to CMH Primary Care Clinics and to explore the relationships between assessment and counseling with child weight status.

Aim 2. To map longitudinal data on BMI, neighborhood environment variables, and other key metabolic health indicators from CMH primary care clinic records to show concentrations of overweight/obesity in the KC region.

Aim 3: To examine the association between physical activity, sedentary behaviors and cardiometabolic health indicators and to examine if these associations are similar across all BMI classifications.

BACKGROUND

Childhood obesity is epidemic. Children who are obese are likely to continue to be obese, which leads to higher risk of obesity related co-morbidities throughout the lifespan. Childhood overweight and obesity affect 4 in 10 children seen in the Children’s Mercy Primary Care Clinics. Although primary care provider (PCP) recognition of this issue is increasing, effective treatment modalities are rarely offered in the primary care setting. In 2012, improved methods of assessing nutrition and physical activity behaviors associated with weight status (Healthy Lifestyle Screen) and provider counseling (Healthy Lifestyle Plan) on these efforts was instituted. A continuous quality and process improvement (CQPI) project was launched in 2014 to provide a means for ongoing staff and provider support and improvement of the HWS and HWP process.

RATIONALE

Although overall clinic rates of HLS performance and provider HLP provision have been tracked (with the assistance of Medical Information Technology) to inform the CQPI project direction, neither individual patient responses to HLS questions nor the HLP that patients received has been studied.

The maps and aggregated health information (by zip code, neighborhood, census track, and other levels of aggregation) will be posted on the Center for Children’s Healthy Lifestyles & Nutrition’s and/or Weighing In website. CMH staff and community organizations will be able to use this information to target their efforts and to utilize in grant applications.

Aim 3 rationale: Engaging in more physical activity and less sedentary behavior is essential as it relates to positive health outcomes. Examining these behaviors in children and their relationship to cardiometabolic indicators is especially important as they have life-long health implications. There is limited researching looking at the relationship between physical activity/sedentary behavior, health indicators, and BMI classification in children.

STUDY DESIGN

This study will consist of a retrospective review of demographic and clinical data obtained from the medical records of patients seen in the CMH Primary Care Clinics between the dates June 1, 2012 and March 31, 2019*.*

1. TARGET STUDY POPULATION SPECIFICS

The population to be studied consists of children ages 2 and older and adolescents presenting for a well child check (WCC) to the CMH Primary Care Clinics during the study period. The total number of patient records to be examined is estimated to be 69,000.

- 1. Inclusion Criteria
- *Patient seen at CMH Primary Care Clinics between the dates June 1, 2012 and March 31, 2019 for a WCC*
- *Patient is age 2-18 years at time of initial visit*
- *Patient’s medical record is available*

Exclusion Criteria

- *Patient under the age of 2 years or over the age of 18 years*
- *Patient visit other than a WCC.*
- *Patient’s medical record is unavailable*

1. DATA COLLECTION

Data Collection Procedures

CMH Investigators will ask Medical Information Technology (MIT) to abstract clinical and healthcare utilization data from subjects’ medical records. MIT will create a dataset that includes data points for primary measures at each visit to the CMH Primary Care Clinics during the period of interest.

Records to be kept

The data on primary measures will be provided by CMH MIT. The information will be stored in an Excel spreadsheet. Each subject will be assigned a unique study identification number (study ID). A master ID list will be maintained that links the subject’s medical record number, date of birth, and address with their study ID. The medical record number, date of birth, and address will be removed from the research database containing the protected health information. Since this research will serve as a baseline for continuing quality improvement, dates of service are necessary to be present in the study database temporarily to ensure accuracy when merging new data with the existing research record and coding data. The master ID list will be destroyed upon study completion.

Participants’ addresses will be geocoded using the US Census Bureau Geocoder (<https://geocoding.geo.census.gov/geocoder/>) to identify the census block group each participant resides in. Then the census block group ID number will be sent to MARC (Mid American Regional Council) to generate census block group centroid geocoordinates and environmental variables for each census block group. The information transmitted to MARC will be de-identified and will not be traceable to participants because it will simply be a list of census block groups in the KC metro area. Next, the centroid coordinates for each census block group will be uploaded into WalkScore (<https://www.walkscore.com/>) to generate WalkScore environmental variables for each census block group (although WalkScore can be obtained for zip codes, more specificity is provided when obtained for census block group centroids. Census block groups within the same zip code often vary with regards to their Walk Score and other environmental variables). The information transmitted to WalkScore will be de-identified and not be traceable to participants because it will simply be a list of block group centroids (generic geocoordinates). The environmental variables will then be merged with the participant data (eg., BMI data) for analyses and retained on the CMH secure server with access only permitted to study personnel. The neighborhood environmental variables are those known to be related to physical activity and nutrition, and thus are important to investigate for their associate with childhood obesity (see environmental variables in the primary measures table). If a child moves during the timespan the environmental analyses focuses on, environmental information will be collected for both census block groups that the child resided in. These participants (“movers”) will be analyzed separately to investigate whether changes in environments relate to changes in body composition.

Secure Storage of Data

The password-protected study database will be stored on the CMH internal server in a restricted assess departmental folder limited to only listed study personnel. The medical record number, date of birth, and address will be deleted from the study databases as soon as data collection has been completed. Another password-protected excel sheet linking the medical record number, date of birth, and address to the study ID will be stored in a separate restricted access folder on the CMH server. This spreadsheet will only be accessible to the PI and Study Coordinator.

1. STUDY DURATION/STUDY TIMELINE

Month 1 (estimated to be July or Aug 2019) – Data abstraction and preparation of 2016, 2017, and 2018 datasets

Months 2-6 (estimated to be Sept-Nov 2019) – Data analysis and report preparation

Months 6-12 – data publication and dissemination

Aim 3: Month 1 (estimated July /August 2020)  - data abstraction & preparation of datasets from 2012-2019.

Months 2-6 (estimated Sept /Oct 2020 – March/April 2020) Data analysis and report preparation

Months 6-12 - data publication

1. STATISTICAL CONSIDERATIONS

Primary Measures

1. Medical record number (will be replaced in the study database with a study identification number but must initially be provided in reports from Information Systems.)
2. Encounter number
3. Date of visit
4. Clinic location of visit
5. Date of birth (will be deleted from study database but will be kept on master list with study ID and medical record number)
6. Age in years
7. Age in months
8. Gender
9. Race
10. Ethnicity
11. Primary language spoken at home
12. Number of parents residing in the home
13. Insurance category
14. Address (will be deleted from study database but will be kept on master list with study ID and medical record number)
15. Height
16. Weight
17. BMI
18. BMI Z score (calculated by study team)
19. BMI percentile (calculated by study team)
20. Healthy Lifestyle Screen answers
21. Type of Healthy Lifestyle Plan ordered and/or documented
22. Environmental variables:

| Census SES | % households earning <$30,000 per year and median household income |
| --- | --- |
| Census Race/ethnicity | % white non hispanic |
| Census Education | % 25+ years old with no HS diploma |
| Land area | Size of census block group, square miles |
| WalkScore | Walkability of census block group |
| BikeScore | Bikability of census block group |
| TransitScore | TransitScore for census block group |
| Connectivity | Number of intersections in census block group |
| Mixed land use | Number of commercial destinations in census block group |
| Residential density | Number of households per sq mile in census block group |
| Parks | Number and square footage of parks in census block group |
| Food access | Number of grocery stores vs. number of fast food restaurants |

1. blood pressure,
2. total cholesterol,
3. LDL cholesterol,
4. HDL cholesterol,
5. triglyerides,
6. ALT,
7. HbA1c

General Design Issues

The measures can be assigned to one of three categories:

1. Demographic measures: Data on primary measures 1-14 should provide us a description of the patient population and allow for identification of any trends and issues of equity, access, etc. Data on the secondary measures will provide in-depth context and better our understanding of the patient population.
2. Outcome measures: Primary measures 15-21 are recommended indicators used to assess and counsel children for promotion of healthy weight.
3. Outcomes measures: lab measures 23-29 are collected from lab tests and are used in diagnoses of cardiometabolic health issues (e.g. type 2 diabetes, dyslipidemia, etc).

Sample size determination

This study is designed to provide a baseline for future clinical quality improvement projects. We will not test any hypothesis, thus will not perform a sample size and power calculation. A sample of N=70993 visits was obtained for the time period June 1, 2012 – May 31, 2016. We estimate an average of 5,000 visit per quarter for the remainder of 2017, bringing the total N to approximately 115993. We will ask MIT to run an aggregate report and raise the sample size in the IRB application prior to requesting identified data for the subsequent time period.

A data pull from 2016 – 2019 was added to the initial data set. We will now pull additional lab data from these same participants for further analyses.

Data Analyses

The analysis will consist of descriptive and correlation analyses. Appropriate parametric and non-parametric tests will be used to examine baseline differences and differences in outcomes between certain groups of patients. Regression analyses will be used to identify significant predictors of outcome variables. Missing data will be excluded. Outliers will be handled on a case-by-case basis depending upon how they affect statistical test results. Mapping will be performed using Tableau and ArcGIS.

1. HUMAN SUBJECTS

Institutional Review Board (IRB) Review and Informed Consent

This protocol, and any subsequent modifications, will be reviewed and approved by the Pediatric IRB at The Children’s Mercy Hospitals & Clinics.

We request a Waiver of Informed Consent and Waiver of HIPAA Authorization. This project entails no more than minimal risks to subjects as this is a retrospective chart review. Participation in this research will not negatively affect the quality of care received by the patient at the PCC. The study database will be deidentified, and the investigators will take precautions to protect confidentiality (see below).

Subject Confidentiality

All records will be kept in a password-protected Excel file on the CMH internal server and maintained for a minimum of three years after the completion of the study. Confidentiality will be maintained through use of a password-protected deidentified study database and a password-protected list that links study ID to two subject identifiers. We believe two is the minimum number of identifiers that we should maintain since we intend to use the deidentified study database as a baseline for quality improvement. Once all data collection is complete the master linking list will be destroyed.

Study Modification/Discontinuation

The study may be modified or discontinued at any time by the IRB as part of their duties to ensure that research subjects are protected.

1. PUBLICATION OF RESEARCH FINDINGS

Results may be presented at pediatric and/or obesity-related annual meetings.

1. REFERENCES
